# Ag- or Cu-modified geopolymer filters for water treatment manufactured by 3D printing, direct foaming, or granulation

**DOI:** 10.1038/s41598-020-64228-5

**Published:** 2020-04-29

**Authors:** Tero Luukkonen, Juho Yliniemi, Harisankar Sreenivasan, Katja Ohenoja, Mikko Finnilä, Giorgia Franchin, Paolo Colombo

**Affiliations:** 1 Fibre and Particle Engineering Research Unit, University of Oulu, Pentti Kaiteran katu 1, Oulu, 90014 Finland; 2 Department of Industrial Engineering, University of Padua, via Marzolo, 9, Padua, 35131 Italy; 3 Research Unit of Medical Imaging, Physics and Technology, University of Oulu, Aapistie 5 A, Oulu, 90220 Finland

**Keywords:** Environmental sciences, Chemistry, Engineering, Materials science

## Abstract

In this work, we compared the main characteristics of highly porous geopolymer components for water treatment applications manufactured by 3D printing, direct foaming, or granulation. Furthermore, different approaches to impregnate the materials with Ag or Cu were evaluated to obtain filters with disinfecting or catalytic properties. The results revealed that all of the investigated manufacturing methods enabled the fabrication of components that possessed mesoporosity, suitable mechanical strength, and water permeability, even though their morphologies were completely different. Total porosity and compressive strength values were 28 vol% and 16 MPa for 3D-printed, 70–79 vol% and 1 MPa for direct-foamed, and 27 vol% and 10 MPa for granule samples. Both the filter preparation and the metal impregnation method affected the amount, oxidation state, and stability of Ag and Cu in the filters. However, it was possible to prepare filters with low metal leaching between a pH of 3–7, so that the released Ag and Cu concentrations were within drinking water standards.

## Introduction

Cellular ceramics have a high potential in water treatment applications, such as Ag impregnated point-of-use disinfecting filters^1^, slurry dewatering systems^2^, catalyst supports^3^, and micro- or ultrafiltration membranes^4^. However, although ceramic materials have good chemical and physical stability, their higher price in comparison to organic polymers has hindered more widespread use in industrial water treatment^5,6^.

Geopolymers, on the other hand, are amorphous, low-calcium, aluminosilicate, ceramic-like materials with structural mesoporosity (i.e., pore diameter 2–50 nm)^7^; good mechanical and chemical stability^8^; and cation-exchange capacity^9^. However, in contrast to conventional ceramics, geopolymers consolidate at (near) ambient conditions without high-temperature sintering. Consequently, geopolymer technology could offer a possibility for clean and low-cost production of ceramic-like filters, possessing better chemical stability, and a longer lifespan than polymeric components.

In the context of water and wastewater treatment, geopolymers have already been investigated for a number of applications, such as adsorbents/ion exchangers, photocatalysts, and membranes, as summarized by Luukkonen *et al*.^10^. Some of the outlined uses require modification of the geopolymers with metals. The proof-of-concept study for the use of geopolymers as antimicrobial materials was performed by O’Connor *et al*., who prepared an Ag^+^-exchanged, halloysite-based geopolymer and applied it successfully to inactivate *Staphylococcus aureus* on an agar plate^11^. Metakaolin or fly ash geopolymers have also been modified with Ag-nanoparticles (AgNPs), and the resulting antimicrobial materials have been proposed to be used in construction^12,13^. Furthermore, Cu^2+^-exchanged metakaolin geopolymers have proved to be a promising antimicrobial material to be used against oyster mushroom hyphae^14^. In (photo)catalytic water treatment applications, the successful impregnation of catalytically active metals/semiconductors (such as TiO_2_, Cu_2_O, Cd) into the geopolymer structure is also crucial^15–17^.

Another important property in many water treatment components is high open (i.e., interconnected) porosity, which can be achieved through different manufacturing methods. Porous geopolymers can be prepared, for example, by additive manufacturing (AM), direct foaming, and granulation-geopolymerization, as summarized by Bai & Colombo^18^. With AM, it is possible to precisely control the pore size, pore-size distribution, pore shape, and pore interconnectivity, with a resolution of a few tens to a few hundred micrometers, depending on the selected manufacturing technology^18^. In contrast, direct foaming produces pores with a random distribution, starting from smaller pore diameters compared to those obtained by AM. Moreover, components with stochastic porosity tend to have a lower strength in comparison to components possessing a more homogeneous porous microstructure, such as the ones produced by AM—important properties, such as permeability and tortuosity, can be designed and more easily optimized in the latter. With a filter bed consisting of granulated geopolymers, the porosity is mainly due to the voids between granules, although the granules contain intrinsic porosity as well^19^.

The purpose of this paper is to study the possibility of using metakaolin-based geopolymer to prepare metal-modified water treatment filters. Metakaolin was selected as a precursor since it does not contain impurities and has more reproducible chemical composition than some frequently used industrial by-product-based precursors (such as fly ashes)^20^. The target is to obtain properties comparable to conventional ceramic pot filters: open porosity, compressive strength, and water permeability of not less than approximately 30%, 1 MPa, and 0.001 cm/s, respectively^21–25^. Different methods for filter preparation are compared: AM (specifically, direct ink writing, or DIW), direct foaming (with anionic, cationic, and non-ionic surfactants), and granulation-geopolymerization. Furthermore, different methods for introducing Ag and Cu (as antibacterial and/or catalytically active metals) into the filter structure were tested. The stability of the metal modification was evaluated at different pH conditions. For the first time, a systematic comparison between 3D printing, direct foaming, and granulation-geopolymerization, and their metal-modification methods is provided.

## Results and Discussion

### Comparison of filter manufacturing methods

**Figure 1:**
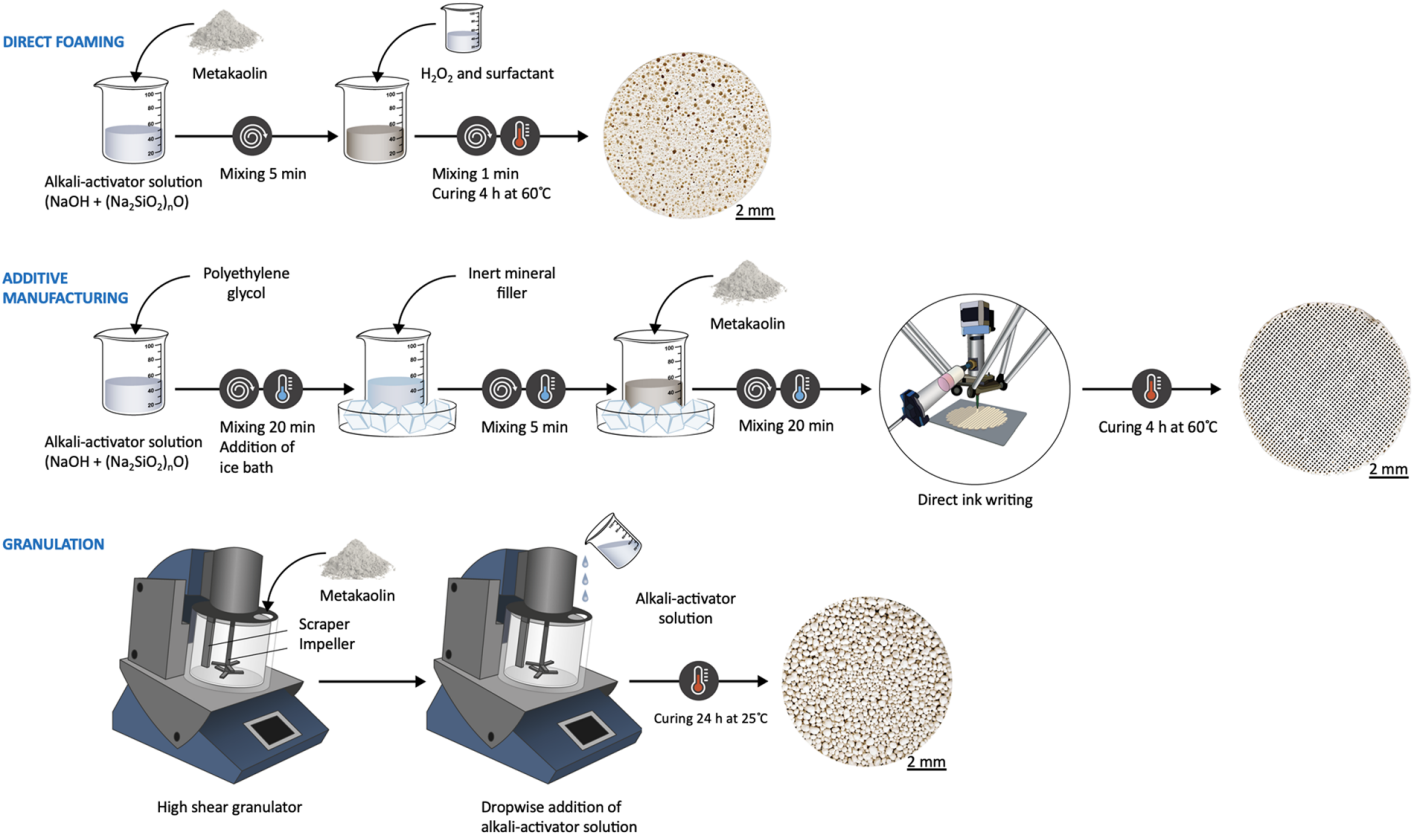
Manufacturing methods and appearance of geopolymer samples. Note that in direct foaming, the solid surfactants were dissolved to alkali-activator solution (i.e., not added together with H_2_O_2_).

Altogether, the filters manufactured by DIW, direct foaming, or granulation-geopolymerization (Fig. 1) all appeared to be suitable for water treatment from the viewpoint of mechanical strength and water permeability. However, the microstructural morphology and pore characteristics of the samples were very different. It should be also noted that geopolymers are dynamic systems meaning that their microstructure may change with time, potentially altering properties required for water treatment. Therefore, microstructure characterization of samples in this study was conducted after one month by which time, most of the changes in the microstructure could be assumed to have already happened.

### Direct ink writing

**Figure 2:**
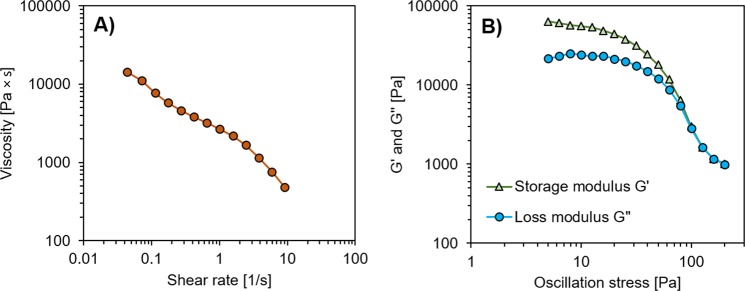
(**A**) Rheological behavior of the paste showing shear-thinning behavior, and (**B**) dynamic mechanical analysis at 1 Hz showing the yield point at 160 Pa.

**Figure 3:**
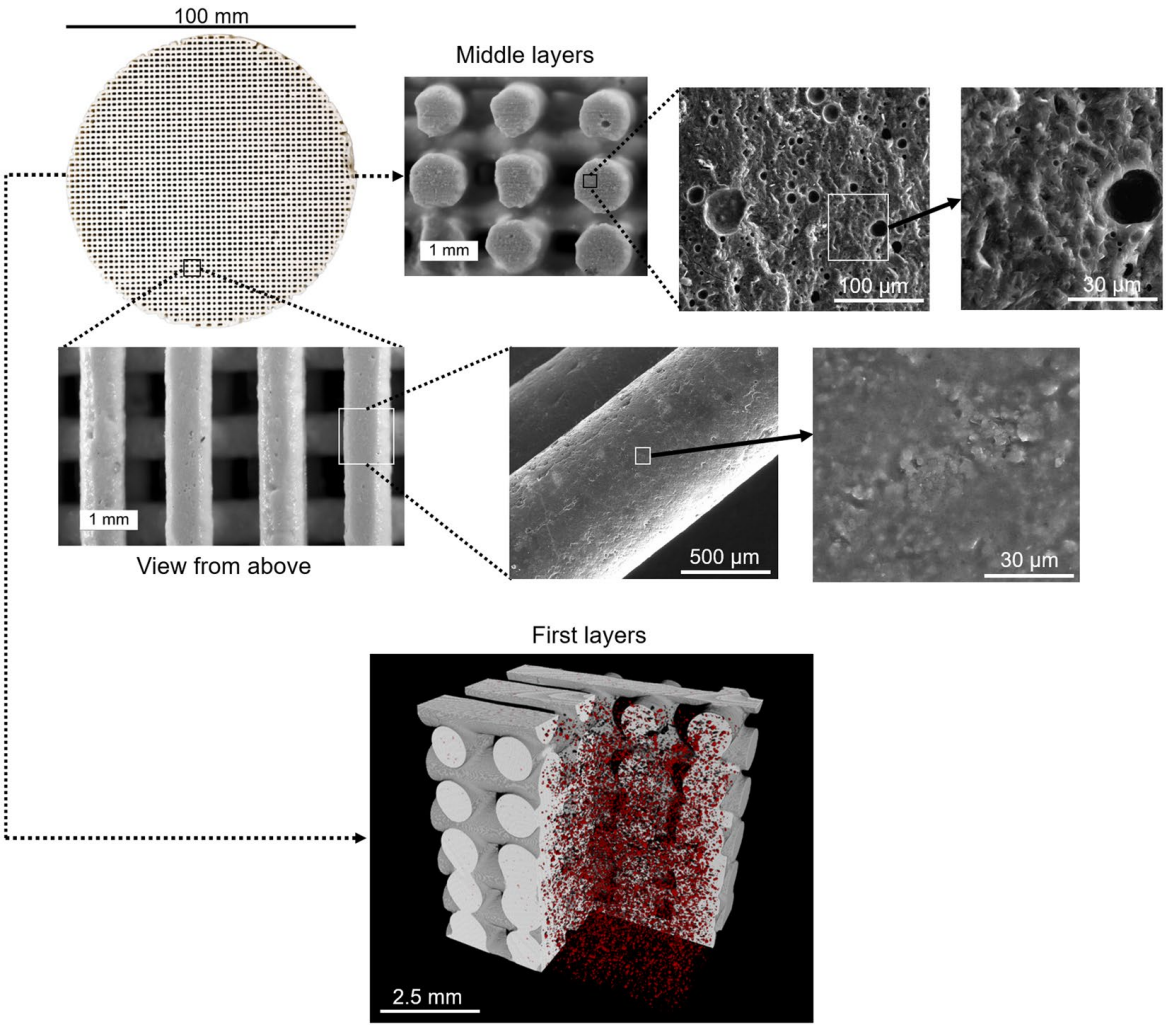
3D-printed geopolymer filter imaged with a DSLR camera, optical microscopy, scanning electron microscopy (SEM), and X-ray microtomography. In the microtomography image, red dots represent the internal (closed) pores, which are also visible in the cross-sections imaged with SEM.

DIW of geopolymers represents technical challenges, as the rheological properties of the paste change as a function of time during the printing process due to on-going polycondensation reactions^26^. In terms of rheology, the aim is to have an ink with a suitable yield stress and shear-thinning behavior. In order for the paste to start flowing, the yield stress value needs to be low enough that it does not require a high pressure for extrusion, but has to be high enough that the structure does not collapse under its own weight after deposition. Also, the setting time of the paste should be sufficiently long enough to enable printing of large components: in the present study, the paste remained printable for 3–4 h. The paste exhibited beneficial shear-thinning (i.e., pseudoplastic) behavior with decreasing viscosity over increasing shear rates (Fig. 2A) and yielding behavior in the dynamic mechanical analysis (DMA; Fig. 2B). It should be noted that the actual shear rates in the extrusion process of geopolymer DIW are likely higher than shown in Fig. 2A; however, the ink behavior is expected to be consistent at higher shear rates, too^26^. In the DMA, at low oscillation stress values (from 1 to 100 Pa), the paste exhibited a linear viscoelastic response (Fig. 2B). The transition out of this region due to the disruption of the formed gel network can be seen as both G’ (storage modulus) and G” (loss modulus) decrease. Using the transition point G’ = G”, the yield point is 160 Pa. This value is in agreement with previous measurements for similar geopolymeric DIW inks^26^. Finally, it should be noted that even though the paste seemed to meet the requirements in terms of rheological properties, the first layers of the scaffolds tended to occasionally sag and merge (Fig. 3). A reason for this could reside in a more pronounced thixotropic character of the paste compared to the ones described in the literature^26^; the raw materials in this work might possess a lower reactivity, and the paste also contains a high amount of filler (i.e., geopolymer particles with similar composition, see Materials and Methods for additional information), so there is less material participating in the gel network formation. As a result, the recovery of viscosity takes too long for the filaments to retain their shape. However, as the condensation reactions continuously progressed with time in the ink, viscosity and yield strength increased and the upper layers of the scaffolds were printed without sagging (as seen in Fig. 3). The difference between the lower and upper layers is remarkable, as it takes quite some time to deposit each layer (∼10 min). In fact, sagging and merging would at least partially decrease the dimension of the openings in the first layers of filaments, reducing the filters’ porosity, surface area, and permeability.

The morphology of the 3D-printed geopolymer filters is shown in Fig. 3. In general, the surface of the filaments appeared to be relatively non-porous, but there were some closed pores within the filaments (∼1 vol%, average diameter of 20 µm), likely due to the difficulty of completely degassing a viscous paste. Higher magnification micrographs (see Supporting Information, Fig. S1) display a high geopolymerization degree (i.e., lack of unreacted plate-like metakaolin particles) and good integration of the filler particles to the gel matrix (no clear interfacial transition zone). When including the volume between filaments, the total porosity of a 3D-printed filter (as determined with X-ray microtomography) varied from 18% (the first layers) to 62% (the upper layers; Fig. 3) with the designed porosity of ∼50 vol%. The total and open porosities determined with the gas pycnometry method were both ∼61%, confirming that X-ray microtomography was a suitable method for analyzing the porosity of 3D-printed samples (i.e., majority of pores were larger than 10 μm). The reason for the low porosity in the first layers is the abovementioned sagging and merging issues. The measured filament thickness (0.79–0.98 mm; Supporting Information Fig. S2) was close to the diameter of the extruder tip (0.84 mm) indicating low shrinkage during drying. The measured separation between filaments in the X-Y direction was 0.5–0.8 mm (0.8 mm corresponded to the designed spacing in the scaffold; smaller separation was measured in the lower layers).

The specific surface area of the 3D-printed material (Table 1) was lower in comparison to direct-foamed or granulated materials. This could be due to the presence of residual PEG-1000 clogging some of the mesopores. Washing with hot, deionized water caused only a limited increase in the specific surface area (from approximately 2 to 6 m^2^/g). The BJH pore size and volume analyses (Table 1 and Supporting Information Fig. S3) also revealed a lower pore volume and larger average pore diameter in printed samples, in comparison to direct-foamed or granulated filters. Most of the pores were located in the mesopore range (2–50 nm), but the proportion of macropores (>50 nm) was larger than that of the other samples (Supporting Information Fig. S3).

**Table 1:** Pore structure, Brunauer-Emmett-Teller (BET)-specific surface area, Barrett-Joyner-Halenda (BJH) pore size, and volume of the different filter materials. Note that microtomography is able to detect pore sizes > ∼10 µm, while the BET/BJH method is able to detect pore sizes <300 nm. Cell wall thickness and average pore width are reported with ± standard deviation.

Sample	X-ray microtomography				BET/BJH			Gas pycnometer method
	Cell wall thickness [mm]	Total porosity [%]	Closed porosity [%]	Average pore width [mm]	Specific surface area [m^2^/g]	Average pore width [nm]	Pore volume [cm^3^/g]	Open porosity [%]^a^
Direct foaming, Triton X-114	0.097 ± 0.026	70.5	1.2	0.558 ± 0.343	36.9	12.3	0.157	85.8
Direct foaming, Triton X-100	0.096 ± 0.026	69.3	0.9	0.504 ± 0.296	35.8	11.6	0.145	84.6
Direct foaming, Triton X-405	0.092 ± 0.026	78.9	1.1	0.763 ± 0.406	22.8	9.3	0.092	84.9
Direct foaming, SDS	0.049 ± 0.013	76.9	0.4	0.276 ± 0.147	21.6	10.5	0.092	86.6
Direct foaming, CTAB	0.071 ± 0.019	71.8	1.0	0.399 ± 0.221	31.0	11.7	0.139	88.8
3D printing	0.79–0.98^b^	62.0^c^ 28.0^d^	2.0	0.5–0.8^e^	6.0^f^	16.9	0.035	60.5
Granulation	—	27.0^g^	4.0	—	27.9	10.2	0.105	62.9^h^

SDS = sodium dodecyl sulfate; CTAB = cetyltrimethylammonium bromide; ^a^ = open porosity ≈ total porosity; ^b^ = filament thickness; ^c^ = porosity in upper layers; ^d^ = porosity in the first layers; ^e^ = diameter of openings between filaments; ^f^ = specific surface area was measured after washing with hot, deionized water (before washing, specific surface area was 2.1 m^2^/g); ^g^ = porosity within granules; ^h^ = porosity of granule bed, including voids between granules.

**Figure 4:**
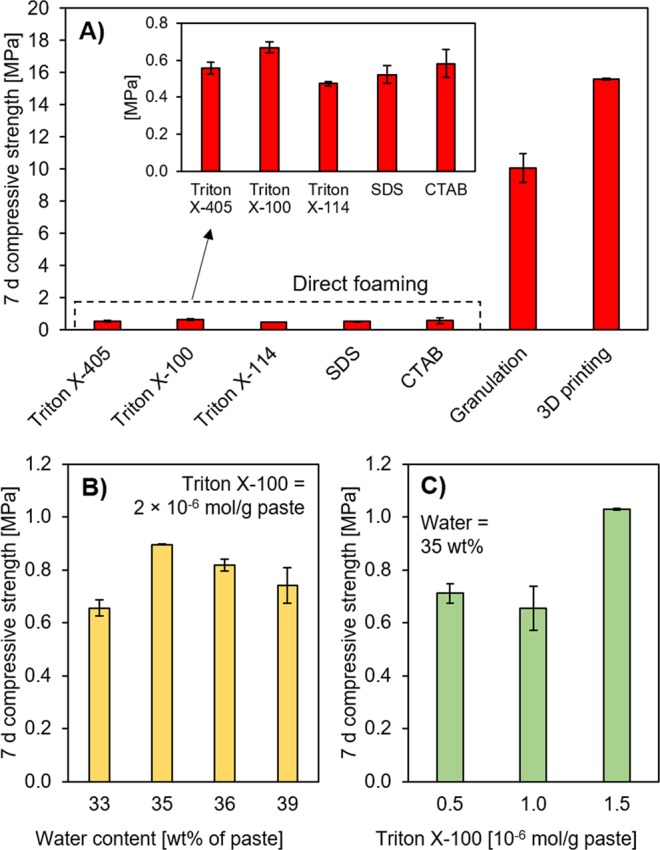
(**A**) Compressive strength (at 7 d age) of filter materials prepared with direct foaming, granulation, or 3D printing. (**B**,**C**) show optimization of water content and surfactant content with Triton X-100. Error bars represent ± standard error. SDS = sodium dodecyl sulfate and CTAB = cetyltrimethylammonium bromide.

The 3D-printed scaffolds clearly exhibited higher compressive strength than direct-foamed or granulated geopolymers (Fig. 4A). The higher strength of the 3D-printed geopolymers can be explained by their lower porosity and thicker structures in comparison to direct-foamed geopolymers (Table 1), and likely a higher degree of geopolymerization reactions in comparison to granulated materials. In terms of the mechanical strength, 3D-printed filters could be most suitable for applications with a high water flow rate, causing higher shear forces.

**Figure 5:**
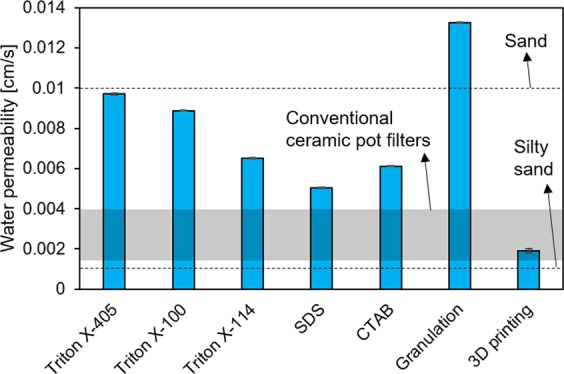
Water permeability coefficients of the filter samples. Values for sand, silty sand, and conventional ceramic pot filters^24^ are shown as reference.

The constant head water permeability coefficient of the 3D-printed filter (Fig. 5) was lower than that of the other samples. This was certainly due to its much lower porosity and to the sagging and merging in the first layers (Fig. 3); moreover, the PEG-1000 plasticizing agent could have increased the surface hydrophobicity in the 3D-printed materials, causing slightly decreased water permeability^27^. Furthermore, the filaments did not contain open porosity, while granulated materials could also be able to provide water permeation by capillary action. This result is, however, somewhat surprising, since the designed filters possessed continuous and unobstructed open channels. This parameter could certainly be increased by modifying the scaffold design and further improving the ink rheology and composition. Nevertheless, the obtained value was still comparable to those of conventional ceramic pot filters^24^.

### Direct foaming

**Figure 6:**
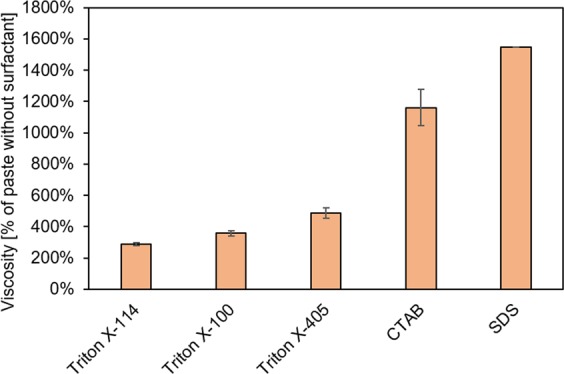
Viscosity of geopolymer pastes containing 1.9 × 10^−6^ mol/g of different surfactants. The results are expressed as a % of the reference paste (without surfactant).

Porosity of the direct-foamed geopolymers was greatly influenced by the viscosity and setting time of the paste, which determined the proportion of generated gas bubbles that were retained in the hardened material. The viscosity of the pastes with different surfactants followed the trend sodium dodecyl sulfate (SDS) > cetyltrimethylammonium bromide (CTAB) » Triton X-405 > Triton X-100 > Triton X-114 (Fig. 6). The clearly higher viscosity values of anionic SDS and cationic CTAB could be explained by interactions with the charged species in the fresh paste (e.g., Na^+^, Al[OH]_4_^−^, H_3_SiO_4_^−^, H_2_SiO_4_^2−^)^28^. Viscosity of pastes containing Triton X-405, X-100, and X-114 increased with molecular weight: Triton X-405 has a longer chain of -CH_2_-CH_2_-O- monomers (Supporting Information Fig. S4), which can have electrostatic interactions with ionic species in the paste. Moreover, a paste viscosity that is too high can inhibit the foaming effect^29^.

**Figure 7:**
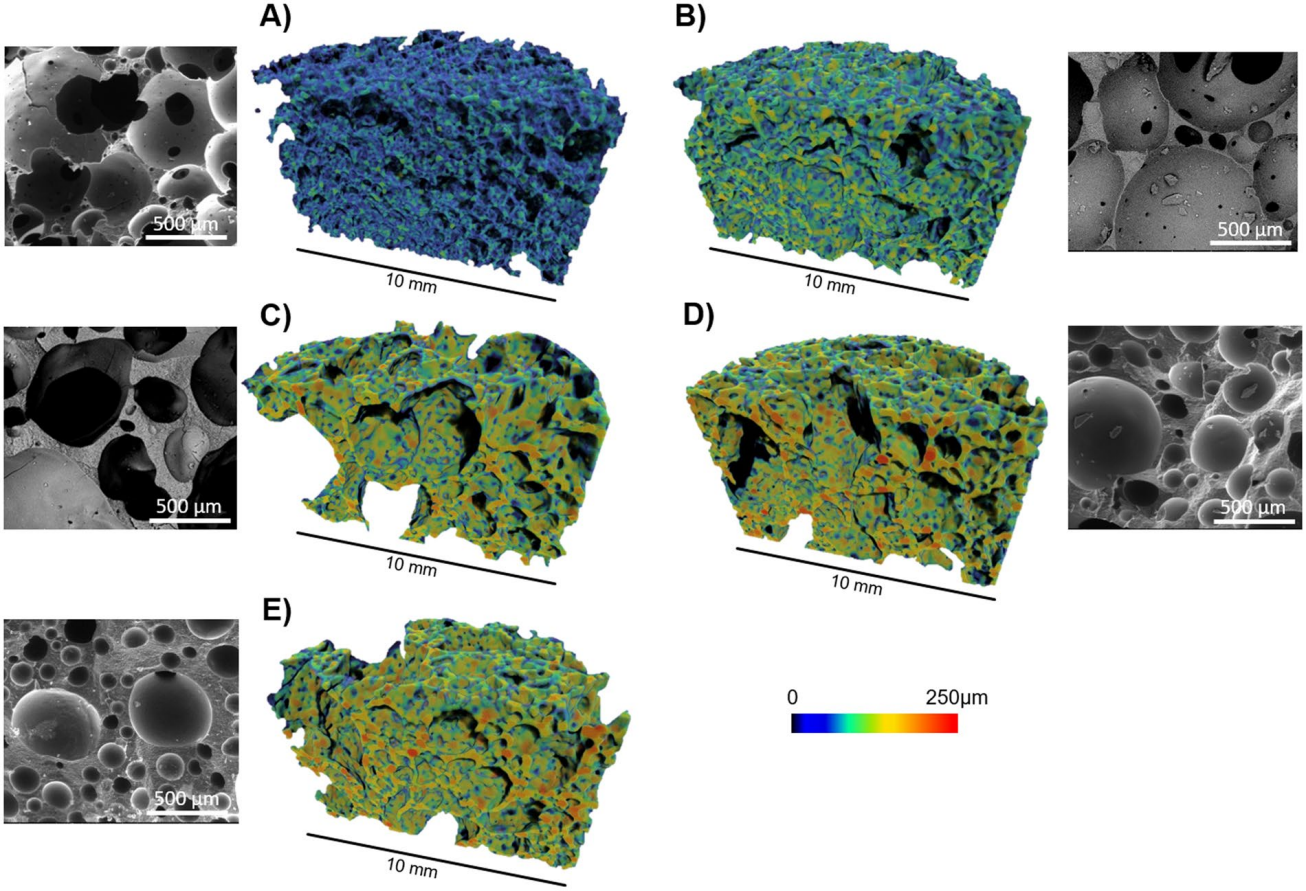
Morphological characterization of direct-foamed samples (with an equimolar concentration of surfactants) in order of ascending cell wall thickness (shown with the color bar): (**A**) sodium dodecyl sulfate (SDS); (**B**) cetyltrimethylammonium bromide (CTAB); (**C**) Triton X-405; (**D**) Triton X-100; (**E**) Triton X-114.

Geopolymer samples produced by direct foaming had a high total porosity (≈71–79%) and the pores were interconnected (i.e., total porosity ≈ open porosity) as shown in Table 1 and Fig. 7. The porosities determined with the pycnometry method (≈85–89%, also in this case total porosity ≈ open porosity, see Table 1) were higher than those obtained with X-ray microtomography indicating the presence of pores smaller than 10 μm. High-magnification micrographs (Supporting Information, Fig. S1) show that there are no unreacted plate-like metakaolin particles present. The width of pore-size distributions (as determined with X-ray microtomography; Supporting Information Fig. S5) followed the trend: Triton X-405 (10–1500 μm) > Triton X-114 (10–1350 μm)> Triton X-100 (10–1150 μm) > CTAB (10–850 μm) > SDS (10–730 μm). The foam prepared with SDS had a high number of pores in a range of 100–350 μm, whereas other surfactants resulted in relatively uniform size distributions. Cell wall thickness values increased as the porosity decreased, with the exception of samples produced using Triton X-405. Specific surface areas of the direct-foamed samples (≈22–37 m^2^/g) were in good agreement with earlier studies (≈17–22 m^2^/g)^9,30^, which indicates that the surfactants used did not clog the nanoscale pores. Singhal *et al*. reported that the addition of CTAB to metakaolin and fumed silica-based geopolymers resulted in a substantial increase in surface area (from 137 to 216 m^2^/g) and suggested that CTAB could act as structure-directing agent during synthesis^31^. Nevertheless, in the present study, no improvement on surface area was observed as a result of using CTAB. The majority of the pores in the direct-foamed samples were located in the mesoporous region, as is typical for geopolymer materials (Supporting Information Fig. S3).

Direct-foamed geopolymers prepared with different surfactants (i.e., anionic, cationic, and nonionic) at an equimolar dose resulted in only small differences in terms of compressive strength (Fig. 4A). Nevertheless, Triton X-100 was selected for further experiments since it resulted in a slightly higher strength. The resulting paste viscosity was still appropriate for the foaming process, and this surfactant did not require a cumbersome dissolution step, as in the case of SDS or CTAB. When the water and surfactant amounts were optimized (Fig. 4B,C), it was possible to increase compressive strength by approximately 50% (to ≈1.0 MPa). The observed optimum in the water content (Fig. 4B) is likely a combination of the effects on the gas-bubble retainment within the paste (i.e., decreased viscosity of paste), hydrogen peroxide decomposition kinetics (i.e., dilution of activator), and extra water acting as a pore-forming agent itself^32^. The amount of Triton X-100 also exhibited an optimum in terms of mechanical strength (Fig. 4C), which could be related to the minimum concentration to reach minimum surface tension (i.e., maximized foaming effect)^29^. Further increase in the concentration led to increased viscosity, which can prevent foaming^29^. For comparison, the compressive strength of some sintered ceramic water treatment filters with similar porosity have been reported in the range of approximately 0.9–1.7 MPa^21–23^. To obtain higher strengths, the porosity should be decreased^33^.

Direct-foamed materials prepared with non-ionic surfactants (especially Triton X-405 and Triton X-100) would allow for a high hydraulic conductivity, comparable to a sand filter (Fig. 5). It has been noted that conventional ceramic pot filters are prone to clogging by suspended matter, and thus high water flows would be beneficial^34^. However, the mechanical strength of the direct-foamed materials could limit their use in high-flow (i.e., high shear force) applications.

### Granulation-geopolymerization

Granulation-geopolymerization differs from AM and direct foaming, since a bed of individual granules is obtained (see Fig. 1) instead of a porous monolith. The formation mechanism is also different as alkaline solution is slowly dosed to the metakaolin powder bed and mixed with an impeller. First, the liquid wets and joins the particles together, then it dissolves the metakaolin, and finally a new aluminosilicate gel (geopolymer) begins to form^35^. When the dosing of the alkaline solution ends, metakaolin particles are still bound to the wet surface of the granules, forming a less dense surface layer^19^.

**Figure 8:**
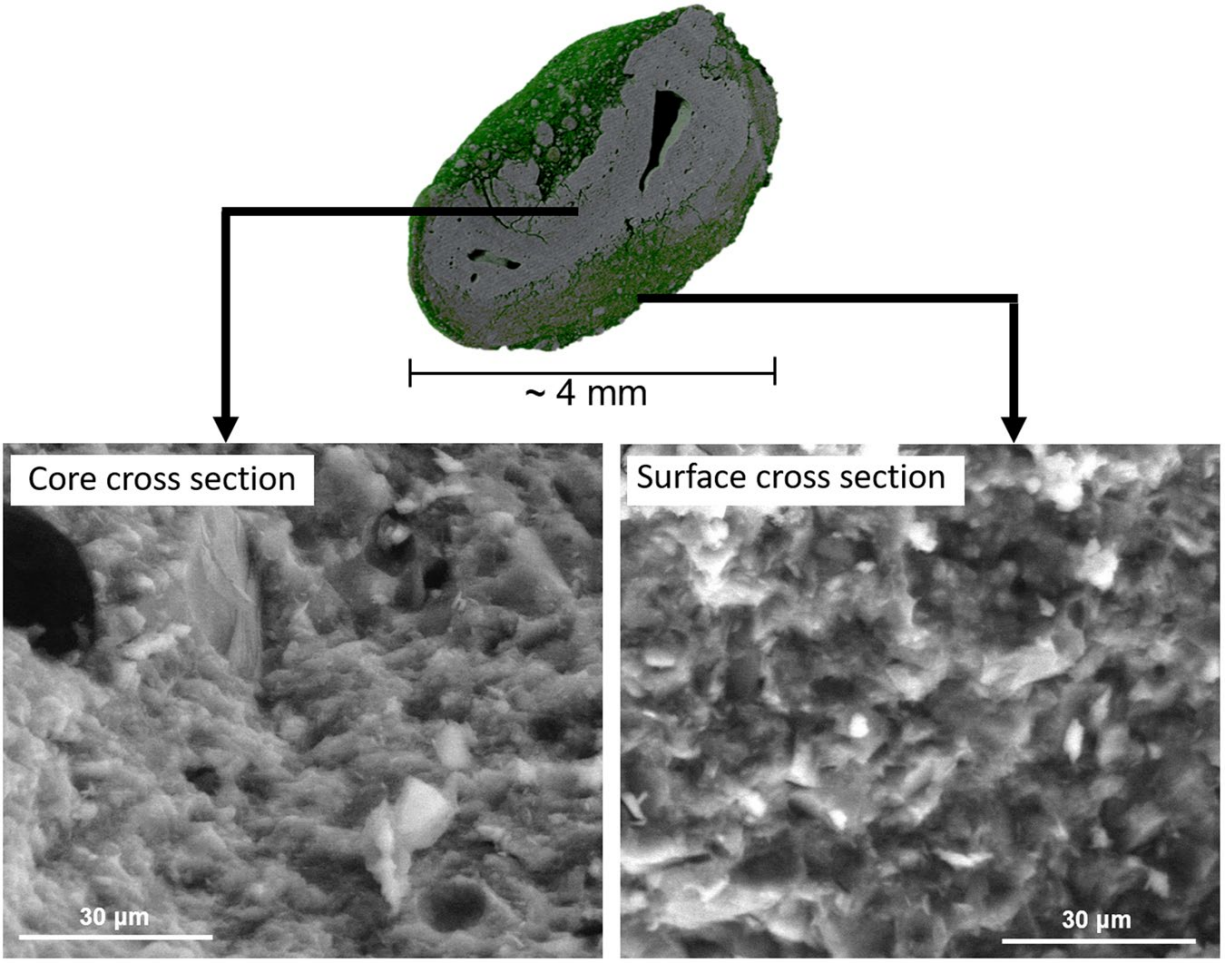
Morphological characterization of a geopolymer granule with X-ray microtomography and SEM. The green part represents the porous surface layer and the gray part represents the dense core.

As mentioned above, granulated geopolymers consist of two phases: a low-density surface layer (volume proportion of approximately 38%) and a dense core (35%; Fig. 8). However, the high-magnification micrographs (Fig. 8) reveal no clear differences in the microstructure of core and surface layer. The total porosity of the granules was 27%: most of the porosity was found at the surface layer, whereas the porosity of the core was only about 4 vol%. The total porosity of the granule bed (1–4 mm) was 63%, including the voids between the granules. Furthermore, the structure of the granules suggests that the formation likely occurs through a surface-layering mechanism.

Granules have a combination of high compressive strength (Fig. 4A) and very high water permeability (Fig. 5). This makes their use ideal for situations requiring high flow rates. Moreover, granules are easy to replace in a filter once their useful life has been reached. In fact, the suitability of geopolymer granules for wastewater filtration (ammonium adsorption) was demonstrated in a small bench-scale pilot test (flow rate 12 L/h) at a wastewater treatment plant lasting approximately three days: the granules did not show any physical degradation during the test^19^.

### Modification of filters with Ag or Cu

Both the filter preparation method and the metal addition method had an impact on the metal content and oxidation state, as shown in Table 2. The mechanisms of metal fixation to the filter material were different depending on the filter structure and the impregnation method of Ag or Cu. The targeted amount of Ag or Cu in the filters was 0.05% (500 mg/kg), based on commercial Ag-modified filters^36^, when Ag-nanoparticles (AgNPs), AgNO_3_, or Cu(NO_3_)_2_ were added to the fresh geopolymer paste. The obtained Ag or Cu content with these methods was only 15–75% of the target value, indicating that part of the metals could have been leached out by the water removal during the consolidation process^37^.

**Table 2:** Ag and Cu content and oxidation state in the modified filters, as determined by X-ray photoelectron spectroscopy (XPS) analysis. Metal content is expressed as an average of three measurements ± standard deviation.

Material/preparation method	Modification method	Ag [mg/kg]	Oxidation state of metal	
			Ag(0) [%]	Ag(I) [%]
AgNPs	—	19300	94	6
3D printing	Addition of AgNPs	127 ± 12	25	75
Direct foaming	Addition of AgNPs	117 ± 38	42	58
Granulation	Addition of AgNPs	76 ± 0.6	55	45
3D printing	Dipping in colloidal Ag	123 ± 15	68	32
Direct foaming	Dipping in colloidal Ag	133 ± 21	77	23
Granulation	Dipping in colloidal Ag	225 ± 30	82	18
3D printing	Addition of AgNO_3_	127 ± 15	0	100
Granulation	Addition of AgNO_3_	74 ± 0.6	0	100
Direct foaming	Ion exchange	110 ± 10 ^a^ 73 ± 6 ^b^	0	100
Granulation	Ion exchange	83 ± 2 ^a^ 89 ± 6 ^b^	0	100
		Cu [mg/kg]		
			Cu(I) [%]	Cu(II) [%]
3D printing	Addition of Cu(NO_3_)_2_	350 ± 0	5	95
Granulation	Addition of Cu(NO_3_)_2_	377 ± 75	3	97
3D printing	Ion exchange	13300 ± 1752 ^a^ 42000 ± 14171 ^b^	5	95
Granulation	Ion exchange	25967 ± 2919 ^a^ 45467 ± 709 ^b^	4	96
Direct foaming	Ion exchange	66500 ± 4959 ^a^ 61133 ± 1457 ^b^	4	96

AgNPs = Ag nanoparticles; ^a^ = ion exchange from Na^+^ to Ag^+^/Cu^2+^, ^b^ = ion exchange from Na^+^ to NH_4_^+^ and then to Ag^+^/Cu^2+^.

When metal salts, AgNO_3_ or Cu(NO_3_)_2_, are added to the fresh geopolymer paste, Ag^+^ and Cu^2+^ precipitate as brown Ag_2_O (at pH > 9) or blue Cu(OH)_2_ (at pH > 8), respectively (this was seen by the color change of the filters; see supporting Information Fig. S6)^38^. Precipitated metal ions were then physically trapped inside the geopolymer structure or they could isomorphically substitute Si or Al in the aluminosilicate network^39^. AgNO_3_ or Cu(NO_3_)_2_-modification was not possible when using the direct foaming method, since the presence of transition metals decomposed hydrogen peroxide too quickly and no foam was formed.

AgNPs contained Ag^0^ supported on bentonite. Bentonite can be incorporated into the geopolymer structure as filler^40^, but it can also react in alkali activation to some extent^41^. Approximately 50–75% of Ag^0^ in AgNPs was oxidized to Ag(I) as indicated by the XPS analysis (Table 2). Both of the Ag oxidation states can, however, provide antimicrobial properties for filters^42,43^.

The mechanism of Ag introduction with a colloidal Ag solution likely consisted of initial absorption of the solution by the porous material followed by adsorption of Ag^0^ on the geopolymer surface, similarly as with conventional ceramic filters^44^. The highest amount of Ag deposited using the dipping method was obtained for geopolymer granules. Dipping into a colloidal Ag solution resulted in a higher content of Ag^0^ in the filter material compared to the addition of AgNP to the fresh paste (Table 2).

The ion exchange in geopolymers occurs on the negatively charged [AlO_4_]^5−^ tetrahedral interlayer and surface sites in the aluminosilicate network^45^. The initial ion exchange of Na^+^ to NH_4_^+^ has been reported to be beneficial for subsequent ion exchange steps with, for instance, Ti species or Co(II)^9,17^ (although in some cases it has also decreased subsequent ion-exchange efficiency^11^). One possible explanation for this could be the increased specific surface area of geopolymers after Na^+^ to NH_4_^+^ exchange^46^. In the present study, the initial NH_4_^+^ exchange decreased or did not affect the subsequent Ag^+^ ion exchange and increased or did not affect the Cu^2+^ exchange (Table 2). The amount of Cu in the ion-exchanged geopolymers was 1.3–6.7 weight-%, whereas Ag reached only maximum 0.1 weight-%. The highest reported ion exchange capacity of geopolymer for Cu is 152 mg/g (15.2 weight-%) in the literature^47^. The better ion-exchange efficiency of Cu is likely due to its higher charge and the smaller aqueous radius (Ag^+^ 0.102 nm vs Cu^2+^ 0.072 nm) allowing it to better reach interlayer pores^48^.

**Figure 9:**
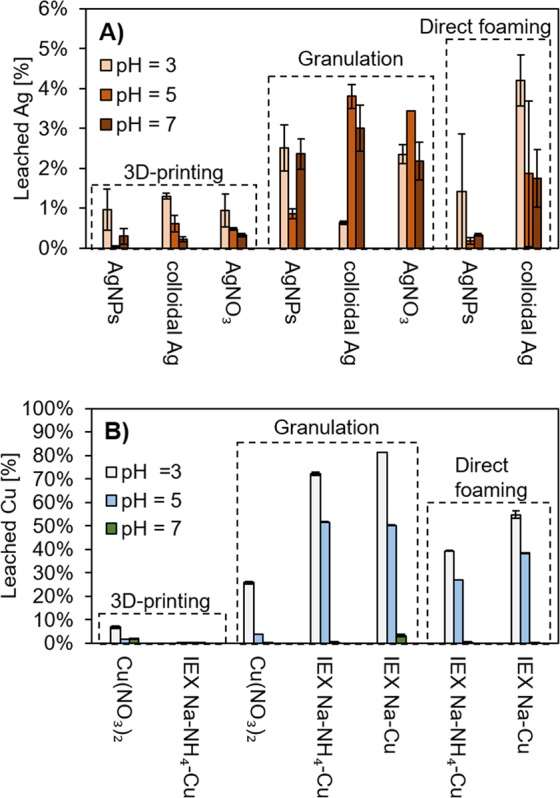
Batch leaching results for (**A**) Ag and (**B**) Cu at an initial pH of 3, 5, or 7 with differently prepared and modified geopolymer filters. For Ag, all ion-exchanged (IEX) materials released 100% of the metals. For Cu, 3D-printed material with Na^+^ to Cu^2+^ ion exchange released 100% of the metals. These results were excluded from the figure. Direct foaming with the addition of a metal salt was not possible due to a catalyzed decomposition of hydrogen peroxide.

The stability of Ag or Cu-modified filters at different pH conditions is reported in Fig. 9. Ag-exchanged materials and 3D-printed Na^+^ to Cu^2+^-exchanged materials are excluded from the list, as metal leaching was approximately 100% (i.e., the metal was leached out almost completely). As expected, a higher extent of leaching was encountered with the lower pH. In the case of Ag, the highest stability was achieved with DIW. This could be explained by the low surface porosity of the 3D-printed materials (see Fig. 3): Ag added to the fresh paste remained effectively entrapped inside the material. On the other hand, however, a large proportion of the Ag is likely not accessible when the filter is used in water treatment. With granules and direct-foamed samples, the higher porosity (see Figs. 7 and 8) caused more contact between the leaching solution and the filter surface, causing a higher amount of metal to be released.

Even though the leaching solutions were buffered, geopolymer materials induced up to a 4.9 unit increase in pH (Supporting Information Fig. S7) due to their residual alkalinity. The extent of the pH increase followed the trend: 3D-printed material> direct-foamed> granulation. However, the increase in pH is expected to decrease quickly when the filter materials are used with continuous flow of water^19^. The initial pH increase and metal leaching could be also decreased with a mild heat treatment, which stabilizes the microstructure^49^.

Finally, an important aspect to consider is the comparison of the released Ag or Cu concentration against the maximum recommended drinking water concentrations, which are 0.1 and 2 mg/L, respectively^50^. In the case of Ag, 3D-printed geopolymers modified with AgNPs, colloidal Ag solution, or AgNO_3_ and direct-foamed geopolymers modified with AgNPs were able to fulfil the guideline. For Cu, on the other hand, only granulated geopolymers with a Cu(NO_3_)_2_ addition leached <2 mg/L of Cu when pH ≥ 5. However, it should be noted that the leaching results represent the initial release of metals (i.e., maximum leaching), which decreases in dynamic flow conditions as a function of time (and therefore a rinsing step before using the filters could be applied).

## Conclusions and Future Research

Direct ink writing is technically the most complicated manufacturing method, as it requires dedicated equipment and accurate control of paste rheology, although it offers several important potential advantages, such as the possibility of morphological optimization. The printed filters displayed the highest compressive strength (≈ 16 MPa), but the lowest water permeability. This was due to the low total porosity (28%) in parts of the filter (despite the open vertical channels present in the filter design) caused by sagging and merging in the first layers of filaments and by the lack of open porosity within the filaments. The specific surface area and pore volume were also lower than those of other materials, probably due to PEG-1000 clogging some of the mesopores. Therefore, the improvement of the rheological properties of the paste, together with the selection and removal of plasticizer, represent further issues to consider.

Direct foaming is a relatively simple manufacturing method, since only a mixer, blowing agent, and surfactant are required. The selection of surfactant (anionic, cationic, or non-ionic) clearly affected the viscosity of fresh paste and, consequently, the morphology of hardened sample. However, the selection of surfactant had little effect on the mechanical strength. The obtained porosity was high (71–79%), with interconnecting pores resulting in high water permeability. Overall, Triton X-100 proved to be the most promising surfactant, and, by optimizing the water and surfactant content, the compressive strength could be increased up to 1 MPa, which is acceptable for water filtration applications.

Granulation-geopolymerization is perhaps the easiest manufacturing method to upscale, and a granule bed is easy to replace after its useful life time. The obtained granules had a porous surface layer and a dense core. The porosity within the single granules and in the granule bed were 27% and 63%, respectively. Granules had a combination of high compressive strength (10 MPa) and water permeability, indicating their high potential for use in water treatment.

The lowest level of Ag leaching was observed with the 3D-printed geopolymer scaffolds modified with AgNPs, dipping into a colloidal Ag solution, or the addition of AgNO_3_ to a fresh paste, and with direct-foamed geopolymer modified with AgNPs. With these manufacturing methods, it was possible to meet drinking water guidelines of a maximum 0.1 mg/L Ag. For Cu, the lowest level of leaching (Cu < 2 mg/L) was reached with geopolymer granules with the addition of Cu(NO_3_)_2_. Ion exchange proved to produce unstable materials that leached 100% of added metals.

The next steps in this research will be to test the most promising filters in water treatment, namely in disinfection and advanced oxidation processes (AOPs). The disinfection process utilizes Ag- or Cu-modified filters in point-of-use water treatment similarly to conventional ceramic pot filters. With AOPs, on the other hand, a small dose of oxidant (such as hydrogen peroxide or other peroxides) will be applied upstream from the filter to generate highly reactive radicals for the mineralization of micropollutants or inactivation of recalcitrant viruses and protozoa, for instance.

## Materials and Methods

### Materials

Metakaolin (MetaMax, BASF; 53.0 weight-% SiO_2_ and 44.5 weight-% Al_2_O_3_) was used as an aluminosilicate precursor. The alkaline solution was prepared by mixing a sodium silicate solution (Merck; molar SiO_2_/Na_2_O ≈ 3.5, water content ≈ 64 weight-%) with sodium hydroxide pellets (VWR Chemicals) to result in a molar ratio of SiO_2_/Na_2_O = 1.2.

Hydrogen peroxide (Honeywell; 30%, w/v) was used as a blowing agent in the direct foaming method. Surfactants employed in the study were Triton X-114 (Acros Organics; 100% solution), Triton X-100 (Sigma-Aldrich; 100% solution), Triton X-405 (Sigma-Aldrich; 70% solution), sodium dodecyl sulfate (SDS; Sigma-Aldrich; ≥98.5 weight-% solid), and cetyltrimethylammonium bromide (CTAB; AMRESCO; ≥ 99 weight-% solid; Supporting Information Fig. S4 for additional information). Polyethylene glycol (PEG-1000; Merck) was used to control the rheology in 3D printing.

Metal modification was conducted using Cu(NO_3_)_2_·3H_2_O (VWR Chemicals; ≥97 weight-%) and AgNO_3_ (VWR Chemicals; ≥99.5 weight-%); bentonite modified with metallic Ag (Ag^0^) and zinc pyrithione (ArgiBlock 001.ZnPy, Laboratorios Argenol; Ag content 1.93 weight-%, d_50_ = 8.12 μm); and colloidal Ag solution (Laboratorios Argenol, Ag content 3051 ppm). The colloidal Ag solution consisted of Ag^0^ particles suspended in water.

### Manufacturing of the filters

The mix design was selected to represent the typical composition^51^ of Ca-free geopolymers (i.e., consisting of sodium aluminosilicate hydrate, or N-A-S-H, gel^52^). The geopolymer had the following molar ratios: SiO_2_/Al_2_O_3_ = 3.24, Na_2_O/SiO_2_ = 0.31, Na_2_O/Al_2_O_3_ = 1.00, and H_2_O/Na_2_O = 9.80. The water content of the fresh paste was 33 wt%. The paste was prepared by mixing metakaolin and the alkaline solution in a weight ratio of 1.00:1.36 for 5 min, using a high-shear mixer (speed 3000 rpm).

In the direct foaming, geopolymer paste was prepared as described above. Solid surfactants were dissolved to alkaline solution whereas liquid ones were added together with H_2_O_2_ to the fresh paste. After addition of H_2_O_2_ and surfactants, paste was mixed for 2 min (3000 rpm) and covered samples were placed in 60 °C for 4 h. After that, samples were kept at ambient conditions. In the surfactant comparison experiments, the amounts of hydrogen peroxide and surfactants were fixed as 0.6 wt% and 1.947 × 10^−6^ mol/g of fresh paste, respectively. In the water amount optimization, additional water was added together with hydrogen peroxide and surfactant, resulting in a water amount of 35, 37, or 39 wt%. In the surfactant amount optimization, the water amount was fixed at 35 wt% and the Triton X-100 concentration was 0.5, 1.0, or 1.5 × 10^−6^ mol/g paste.

Granulation-geopolymerization was performed using a high-shear granulator (Eirich EL1): 200 g of alkaline solution was added drop-wise to 300 g of metakaolin during ~20 min while mixing at a speed of 1200 rpm. Granules with a 1–4 mm diameter were separated by sieving, placed in an airtight plastic bag, and kept at room temperature.

DIW of geopolymer scaffolds was performed with a Delta 2040 Turbo (WASP) equipped with the paste extrusion system LDM WASP Extruder (WASP). To prepare the ink, 5 weight-% of PEG-1000 was dissolved in the alkaline solution to act as a yield stress agent^53^. Then, under an ice bath, filler was added and mixing was continued for 5 min (speed 1000 rpm). The filler was metakaolin geopolymer, which was prepared similarly as the ink (except without PEG-1000) and crushed to particle size <300 μm. Optimum filler content was found at 43 weight-% (from the range of 25–65 wt%). Then, metakaolin was added and the paste was mixed for 20 min (speed 3000 rpm). The paste was loaded into a syringe (Nordson EFD) and degassed using a conditioning mixer (ARE-250, Thinky) for 1 min (speed 2000 rpm). The extrusion was performed through a 0.84 mm tip (Nordson EFD). The 3D-printed scaffolds consisted of 10 layers of high disks (Ø ≈ 100 mm) or 16 layers of high square prisms. The scaffolds had a designed porosity of 50 vol%. After printing, scaffolds were covered and placed in 60 °C for 4 h.

### Ag and Cu modification of filters

Ag or Cu was introduced to the filters with a targeted amount of 0.05 weight-% as used in some commercial filters^36^. Ag addition was performed by: 1) dissolving AgNO_3_ in water and adding the solution to the fresh paste; 2) adding Ag^0^-containing bentonite to the fresh paste; 3) dipping the filters for 45 s in 800 mg/L colloidal Ag solution^54^; or 4) ion exchange by immersing the filters in 0.29 M NH_4_Cl for 24 h, flushing with deionized water, drying at 60 °C for 24 h, and immersing in 0.1 M AgNO_3_ for 24 h, followed by similar flushing and drying^17^. Ion exchange was also carried out without the NH_4_Cl step. Cu addition was performed similarly. In geopolymerization-granulation, Ag or Cu salts were mixed with the alkaline solution or the AgNP-containing bentonite was mixed with metakaolin before granulation.

### Rheological properties of geopolymer pastes

The rheological properties of the ink used for DIW were investigated with a Discovery HR-1 rheometer (TA Instruments) with a Ø 40 mm parallel steel plate geometry and a 1 mm gap between the plates. Measurements were conducted after 30 min of ink preparation (at 22 °C). A flow ramp was conducted at shear rates of 0.01–10 s^−1^. Dynamic mechanical analysis (DMA) was carried out at 1 Hz changing the oscillation stress from 0.1 Pa to 300 Pa. The yield stress of the paste was determined by observing the stress at which point G’ was equal to G” in the DMA tests. The viscosity of the pastes containing different surfactants was determined using a DV-II + Pro Extra viscometer (Brookfield).

### Characterization of filters

The compressive strength (at 7 d after preparation) was evaluated using a Zwick/Roell Z10 testing machine with a 1 mm/min loading rate. Samples were 15 × 15 × 8 mm^3^ scaffolds (DIW); 50 × 50 × 50 mm^3^ cubes (direct foaming); and 3.5 mm diameter granules. The granules were selected so that their shape was spherical as closely as possible. Compressive strength (σ, [MPa]) was calculated using Eqs. 1 and 2 for the prisms and granules, respectively (F = peak force [N], A = surface area under compression [mm^2^], and d = diameter of granule [mm]). Equation 2 is based on the standard ISO/CD 11273-2^55^.

1 $${\rm{\sigma }}=\frac{{\rm{F}}}{{\rm{A}}}$$

2 $${\rm{\sigma }}=\frac{4\times {\rm{F}}}{{\rm{\pi }}\times {{\rm{d}}}^{2}}$$

Specific surface area and nanoscale pore volume distributions were determined (1 month after preparation of samples) using N_2_ gas adsorption-desorption (i.e., Brunauer-Emmett-Teller [BET] isotherm and the Barrett-Joyner-Halenda [BJH] method) with Micromeritics ASAP 2020 from crushed samples with a particle size <600 μm.

The constant head water permeability coefficient (k, [cm/s]) was determined with a set-up shown in Fig. S8 (in supporting information). Direct-foamed samples were cast in a plastic column (Ø = 10 cm) used in the measurement and, after consolidation, their top and bottom surfaces were briefly ground using abrasive paper (grade P60) to reveal pores. 3D-printed disc were tightly fitted to the plastic column using polytetrafluoroethylene thread seal tape. Granules (1–4 mm) were placed in the column as a bed. Calculations were conducted using Eq. 3 (η_T_ = water viscosity at the measurement temperature [Pa × s], η_20_ = water viscosity at 20 °C [Pa × s], Q = water flow [cm^3^/s], h = sample height [cm], A = sample area [cm^2^], p = water head [cm]).

3 $${\rm{k}}=\frac{{{\rm{\eta }}}_{{\rm{T}}}}{{{\rm{\eta }}}_{20}}\times \frac{{\rm{Q}}\times {\rm{h}}}{{\rm{A}}\times {\rm{p}}}$$

Open and total porosities were determined with the gas pycnometer (AccuPyc II 1340, Micromeritics, using He gas) method. Geometric density [ρ_g_, g/cm^3^] was determined from oven-dried samples (24 h at 60 °C) using an analytical balance and caliper. Apparent (ρ_a_, [g/cm^3^]) and true densities [ρ_t_, g/cm^3^] were obtained from crushed (maximum dimension approximately <1 cm) and pulverized (<75 μm) samples, respectively, using the pycnometer. Total, open, and closed porosities were calculated according to Eqs. 4–5.

4 $${\rm{Total}}\,{\rm{porosity}}[ \% ]=\frac{{{\rm{\rho }}}_{{\rm{t}}}-{{\rm{\rho }}}_{{\rm{g}}}}{{{\rm{\rho }}}_{{\rm{t}}}}\times 100$$

5 $${\rm{Open}}\,{\rm{porosity}}[ \% ]=\frac{{{\rm{\rho }}}_{{\rm{a}}}-{{\rm{\rho }}}_{{\rm{g}}}}{{{\rm{\rho }}}_{{\rm{a}}}}\times 100$$

High resolution micro-computed tomography scanning (Skyscan 1272, Bruker microCT) was used to determine of porosity and pore-size distribution (at 1 month after preparation). The X-ray tube was set to 50 kV and the generated beam was filtered with a 0.5 mm aluminum filter. Projection images with a pixel size of 3.5 or 4.5 µm were collected every 0.325° over 360° with an exposure time of 2.2 s and a frame averaging of three. Projection images were reconstructed with NRecon (v. 1.6.10.4 using GPUReconServer [1.6.10] as the reconstruction engine, Bruker microCT). To remove noise, the following settings were applied during reconstruction: smoothing (2), ring artifact correction (10), and beam hardening correction (35%). Morphological analysis was performed with CT Analyser software (Version: 1.17.7.2). To keep analysis times reasonably short, a cylindrical volume of interest with diameter a of 1500 pixels and height of 1000 pixels was truncated for analysis. These images were subjected first to median filtering (3D with a radius of 3 pixels). Then, images were binarized with the 3D Ridler-Calvard iterative selection method and subjected to 3D morphological analysis.

Micrographs of the filters were taken with an environmental scanning electron microscope FEI, Quanta 200. Optical microscopy was performed with Leica MZ6 equipped with a camera (Leica DFC420).

X-ray photoelectron spectroscopy (XPS) characterization to determine the oxidation state of impregnated Ag and Cu was carried out using a Thermo Fisher Scientific ESCALAB 250Xi XPS System. C1s (284.6ev) was used as a standard reference. The binding energy of Cu 2p_3/2_ was fixed at 932.2 ± 0.2 eV for Cu(I) and 933.3 ± 0.2 eV for Cu(II), on the basis of the available literature^56–59^. The binding energy of Ag 3d_5/2_ was selected as 378.0 ± 0.2 eV for Ag(0) and 367.2 ± 0.2 eV for Ag(I) according to the available literature^60–62^. Peak fitting was carried out using Thermo Scientific Avantage software for surface analysis. This involved the use of the “Smart” type fitting procedure, sum of Gauss-Lorentz mix, and “Simplex” fitting algorithm.

Triplicate batch immersion tests were performed to evaluate Ag or Cu release^63^. Representative pieces of the filters were placed in buffer solutions with a pH of 3, 5, or 7 (liquid/solid weight-ratio = 15) and agitated using an orbital shaker at 50 rpm for 72 h. Buffer solutions were prepared by mixing 0.1 M potassium hydrogen phthalate (Merck) with 0.1 M HCl (FF-Chemicals) in a volume ratio of 1: 0.446 (pH = 3.00); 0.1 M potassium hydrogen phthalate with 0.1 M NaOH (FF-Chemicals) in a volume ratio of 1: 0.452 (pH = 5.00); or 0.1 M potassium dihydrogen phosphate with 0.1 M NaOH in a volume ratio of 1: 0.582 (pH = 7.00). The Ag or Cu concentration was quantified using an optical emission spectrometer (XSeries II ICP-MS, Thermo Fisher Scientific) following standard methods^64^. The Ag or Cu content of the solids was determined after dissolving with the HNO_3_/HCl digestion^65^.

## Supplementary information

Supplementary Information.

## Data Availability

The raw and processed data required to reproduce these findings are available to download from http://urn.fi/urn:nbn:fi:att:ee1d4793-3c10-4beb-86fe-6db815e3a8cb.
