# Supplementary material for: Ag- or Cu-modified geopolymer filters for water treatment manufactured by 3D printing, direct foaming, or granulation
Source: Sci Rep. 2020 Apr 29;10:7233. doi: 10.1038/s41598-020-64228-5 (PMC7190745; doi:10.1038/s41598-020-64228-5)
Supplement: Supplementary file 1 — Supplementary Information. [file 41598_2020_64228_MOESM1_ESM.docx]

# **Supporting information**

# for

**Ag- or Cu-modified geopolymer filters for water treatment manufactured by 3D printing, direct foaming, or granulation**

Tero Luukkonen^1^, Juho Yliniemi^1^, Harisankar Sreenivasan^1^, Katja Ohenoja^1^, Mikko Finnilä^1^, Giorgia Franchin^2^, Paolo Colombo^2^

^1^ University of Oulu, Fibre and Particle Engineering Research Unit, P.O. Box 8000, FI-90014, University of Oulu, Finland

^2^ University of Padua, Department of Industrial Engineering, via Marzolo, 9, Padova 35131, Italy

Fig. S1. High magnification micrographs of 3D-printed (A and B) and direct-foamed samples (C).


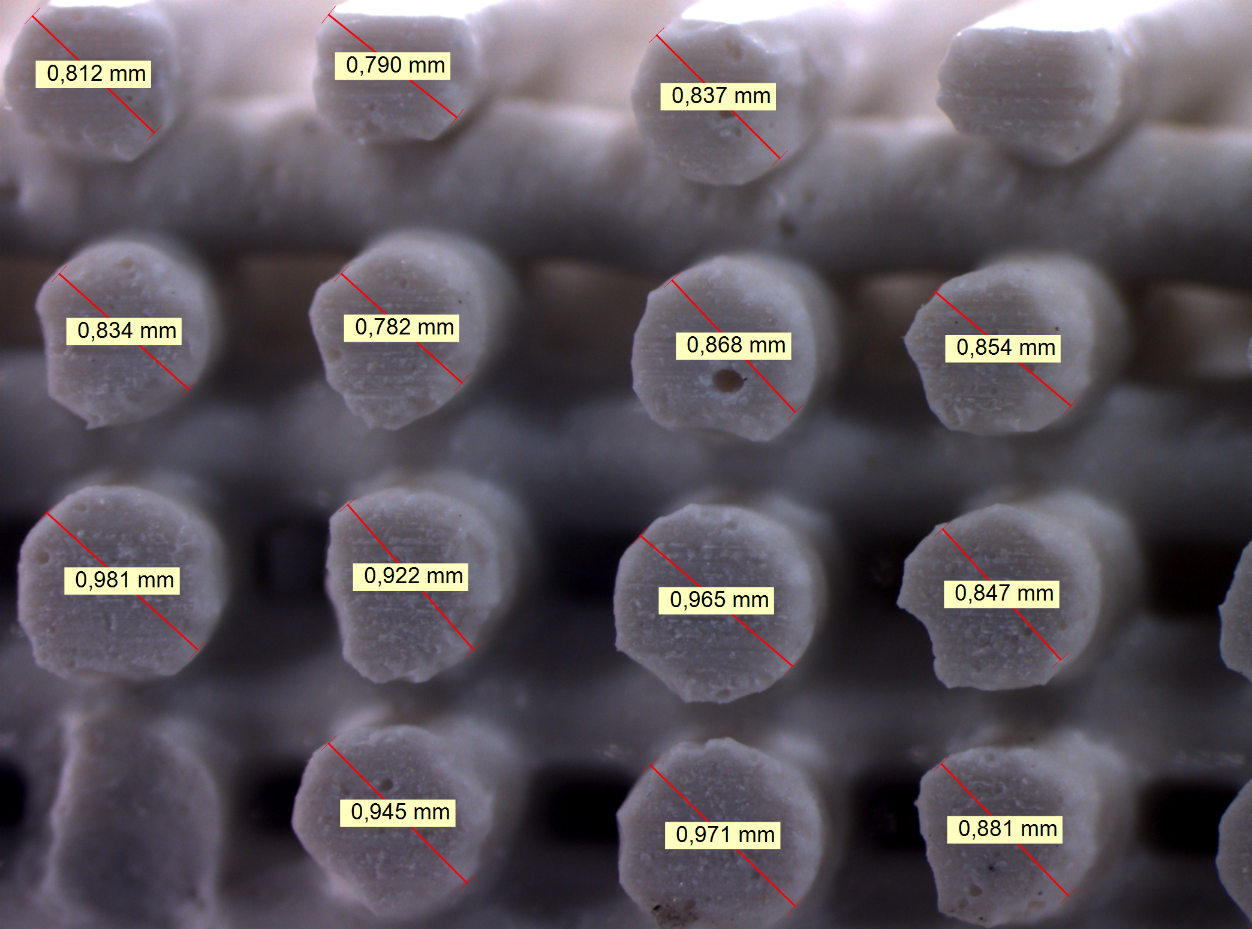


Fig. S2. Diameters of 3D printed filaments determined with a light microscope.

Fig. S3. Cumulative pore volumes of filter materials as a function of pore size determined by the BJH method. Majority of the pore volume is located in 2–50 nm diameter pores (i.e., mesopores).

Fig. S4. Chemical structures and molecular weights of the employed surfactants. Drawn with ACD/ChemSketch, version 2016.2.2 (https://www.acdlabs.com/resources/freeware/chemsketch/).


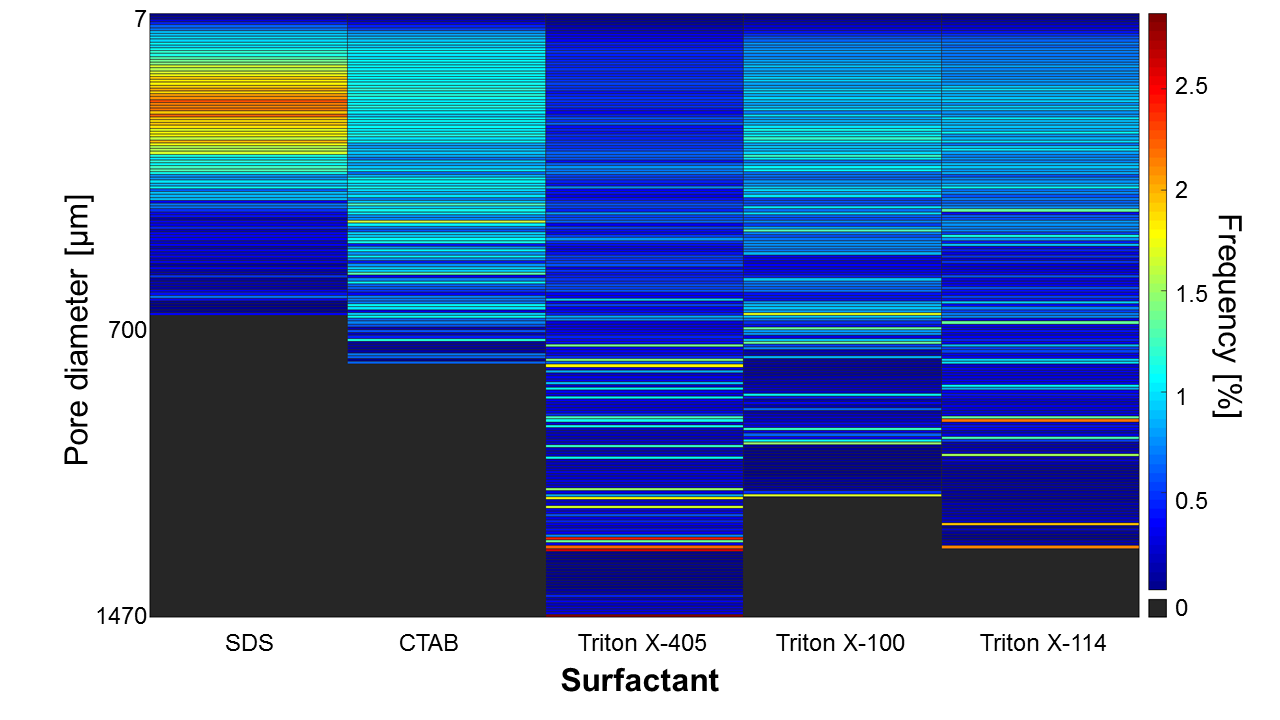


Fig. S5. Pore size distributions of samples prepared with direct foaming (determined with X-ray microtomography).

Fig. S6. Examples of the appearance of filter materials after Ag or Cu modification, A) granules after ion exchange, B) direct-foamed geopolymers after ion exchange, and C) 3D-printed with AgNO_3_ or Cu(NO_3_)_2_ salts added to the fresh-state paste (left picture of each material contains silver and right copper).

Fig. S7. Increase of pH during leaching test with different filter preparation and modification methods.

Fig. S8. The experimental set-up used in permeability coefficient determination.
